# Spot sign as a predictor of hematoma expansion and poor functional outcomes after intracerebral hemorrhage: a systematic review and meta-analysis

**DOI:** 10.1007/s10140-026-02493-z

**Published:** 2026-05-28

**Authors:** Maria Clara Nery Cardoso, Davi Chaves Rocha de Souza, Bruna Rios Beserra, Theo Cardoso Ribeiro, Vinicius Rodrigues Oliveira Alves, Amanda Cerqueira Marcolino, Maria Fernanda Nery Cardoso

**Affiliations:** 1 Bahiana School of Medicine and Public Health, Salvador, Brazil; 2 Federal University of Bahia, Salvador, Brazil; 3 Department of medicine, University of Salvador (UNIFACS), Salvador , Brazil

**Keywords:** Spot sign, Intracerebral hemorrhage, Functional outcome, Hematoma expansion, Mortality

## Abstract

**Purpose:**

The Spot Sign (SS) is a radiological finding on computed tomography angiography (CTA) in patients with intracerebral hemorrhage (ICH). This meta-analysis aimed to evaluate the association between the SS and both hematoma expansion (HE) and functional outcomes by comparing ICH patients with and without SS.

**Methods:**

We searched PubMed, Embase and Cochrane Library for studies of intracranial hemorrhage reporting the SS. The primary outcomes examined were functional status and hematoma expansion, secondary outcomes were mortality and initial hematoma volume. Statistical analysis was performed using RStudio (version 4.5.2), and heterogeneity was assessed with I² statistics. In addition, sensitivity analyses were performed, and publication bias was assessed through funnel plots and Egger’s regression test.

**Results:**

We included 10 observational studies with a total of 2,832 patients with spontaneous ICH. Of these, 613 had SS, while 2,219 did not. The mean age was 66.31 ± 13.81 and 1,367 (58.6%) were male. Poor functional outcomes (OR 4.06, 95% CI 2.66─6.20, *p* < 0.0001, I² = 0.0%) and hematoma expansion (OR 7.17, 95% CI: 5.65─9.09, *p* < 0.0001, I² = 0.0%) were substantially higher in patients with SS, as well as the overall mortality rate (OR 4.14, 95% CI 2.45–6.99, *p* < 0.0001, I² = 65.4%). However, the initial hematoma volume (MD 11.54, 95% CI -11.43─34.52, *p* = 0.2082, I² = 80.1%) was not statistically different between groups. Furthermore, the leave-one-out sensitivity analyses showed that no single study exerted a disproportionate influence on the overall effect for the examined outcomes, and Egger’s linear regression tests were not statistically significant for both primary outcomes.

**Conclusion:**

The presence of the SS is associated with higher rates of poor functional outcomes and hematoma expansion. Therefore, the SS represents a relevant radiological marker with potential to support early risk stratification and optimize patient management and treatment selection.

**Graphical abstract:**

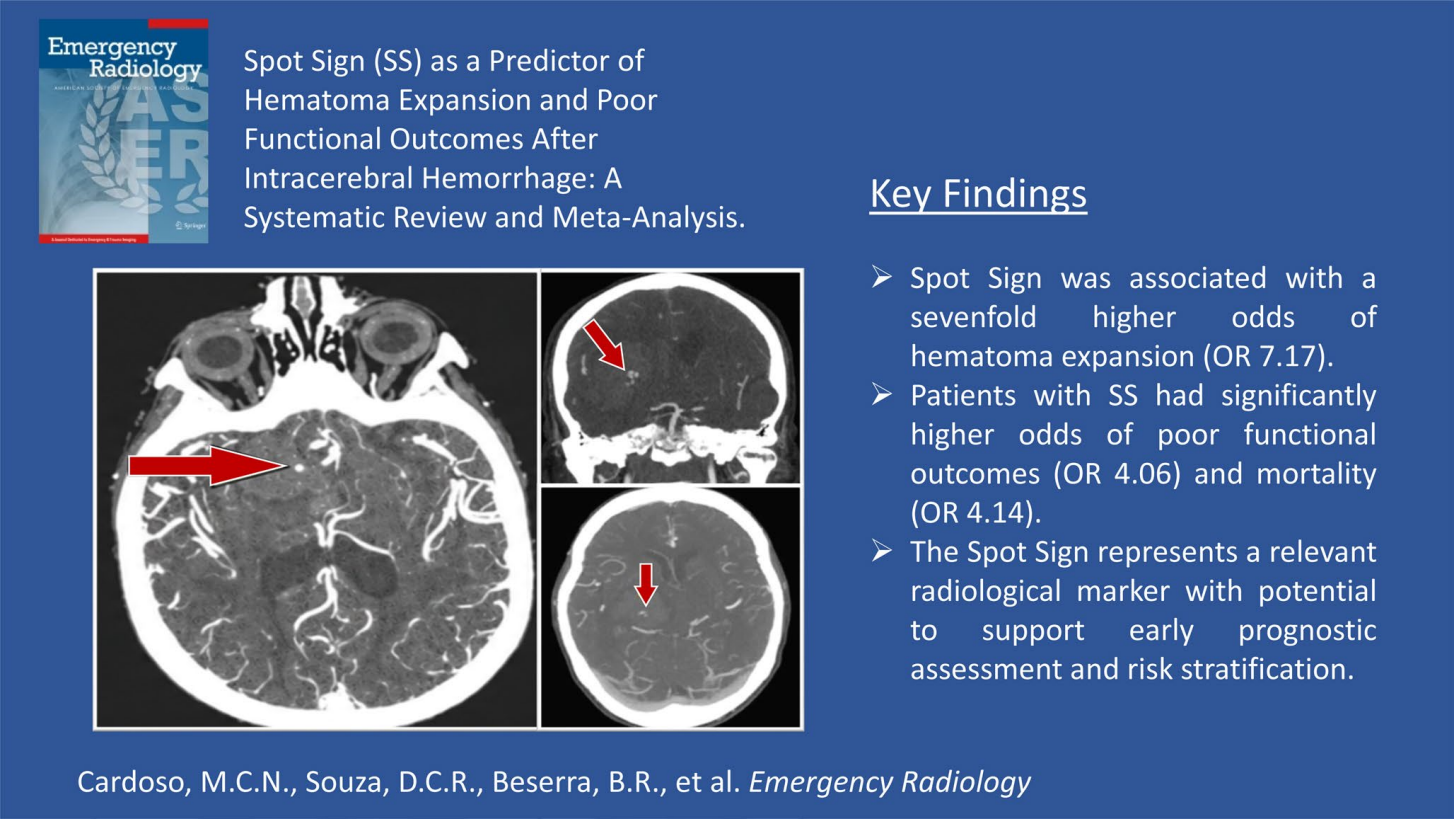

**Supplementary Information:**

The online version contains supplementary material available at 10.1007/s10140-026-02493-z.

## Introduction

Intracerebral hemorrhage (ICH) is a severe life-threatening subtype of stroke, associated with elevated morbidity and mortality rates [1]. Despite advances in acute stroke care, early neurological deterioration continues to be a major determinant of poor clinical outcomes in ICH, driven by hematoma expansion, which represents a critical and potentially modifiable therapeutic target [2].

Therefore, increasing attention has been directed toward imaging biomarkers capable of identifying patients at high risk for hematoma growth. Among these, the computed tomography angiography (CTA) spot sign has emerged as one of the most extensively studied radiological predictors of active bleeding in spontaneous ICH [3]. In this context, the spot sign is characterized by one or more small, contrast-enhancing foci within the hematoma on CTA source images, reflecting contrast extravasation and ongoing hemorrhagic activity [4].

Several observational studies have demonstrated a strong correlation between the presence of the spot sign and subsequent hematoma expansion, as well as worse clinical outcomes, such as increased mortality and unfavorable functional recovery [3, 5]. Previous meta-analyses have further confirmed these associations, demonstrating moderate sensitivity and high specificity of the spot sign for predicting hematoma growth and its correlation with mortality and poor functional outcomes [6–8].

Nevertheless, the most recent comprehensive meta-analyses on the CTA spot sign were conducted several years ago and present important limitations that may affect the interpretation of pooled estimates. Prior studies have demonstrated substantial heterogeneity related to imaging protocols and outcome definitions, which may significantly influence diagnostic performance [6–8].

Since their publication, recent observational studies have provided updated evidence reinforcing the prognostic value of the spot sign. A large multicenter cohort confirmed its role as a strong predictor of severe hematoma expansion [9], while contemporary data have demonstrated its independent association with hematoma expansion and poor functional outcomes [10]. Additionally, analyses incorporating multiple imaging markers have shown that the spot sign remains the most robust predictor of early hematoma growth compared with other radiological features [11]. Together, these findings suggest that incorporating more recent evidence may provide an updated and more comprehensive synthesis of the prognostic value of the spot sign, while reinforcing previously established associations.

Accordingly, although the prognostic value of the spot sign is already well established, an updated synthesis of the available evidence remains warranted to incorporate contemporary cohorts, recent imaging protocols, and additional clinically relevant outcomes that have been less consistently explored in prior pooled analyses. Therefore, the present study aims to perform an updated systematic review and meta-analysis to reassess the prognostic significance of the CTA spot sign in patients with spontaneous intracerebral hemorrhage.

## Methods

### Study design

A systematic review was conducted in accordance with the Preferred Reporting Items for Systematic Reviews and Meta-Analyses (PRISMA) Statement for Reporting Systematic Reviews. The protocol was not prospectively registered with PROSPERO.

### Search strategy

A comprehensive computerized literature search was performed without date limits using the databases PubMed, EMBASE, and Cochrane on December 18, 2025.

The search strategy included terms related to intracerebral hemorrhage and CTA spot sign, combined using appropriate Boolean operators, and was applied across all databases. The full electronic search strategy for each database is provided in Supplementary Table 1.

### Selection process

Study selection was performed in two stages. First, titles and abstracts were independently screened by two authors using Rayyan, according to predefined eligibility criteria. Subsequently, full-text articles were assessed for eligibility. Disagreements were resolved by consensus, with input from a third reviewer.

Inclusion and exclusion criteria were predefined. Eligible studies comprised observational studies (retrospective or prospective cohort studies and case–control studies) published in peer-reviewed journals between 2010 and 2025, in English. Studies were required to include patients with spontaneous intracerebral hemorrhage confirmed by computed tomography (CT) and to evaluate the presence or absence of the spot sign on initial CTA. Studies assessing imaging markers or derived imaging features were also considered eligible, provided that the spot sign was explicitly reported. Additionally, studies were required to report relevant clinical or radiological outcomes, including functional outcomes or hematoma expansion. Studies were excluded if they were non-English, published only as abstracts, lacked outcome data, were case reports or protocols, or did not meet the predefined methodological criteria.

### Data extraction and outcomes definition

Data were extracted independently by three reviewers. Collected data included: (a) first author name and year of publication; (b) study type; (c) study location and period; (d) definition of the spot sign; (e) mean age and standard deviation; (f) number of patients with and without the spot sign and sex ratio; (g) patient treatment for intracerebral hemorrhage; (h) mean follow-up duration; (i) hematoma expansion (HE) and functional outcomes (using the poor outcomes defined by the studies); (j) and secondary outcomes, including overall mortality.

### Statistical analysis

All statistical analyses were conducted using RStudio (version 4.5.2; R Foundation for Statistical Computing, Vienna, Austria). Patients were categorized into the spot sign group and the non-spot sign group. Effect sizes were calculated as odds ratios (ORs) with 95% confidence intervals (CIs) to assess the association between the Spot Sign and hematoma expansion, as well as functional outcome and overall mortality.

Pooled estimates were obtained using inverse variance weighting under a random-effects model, with between-study variance estimated using the restricted maximum-likelihood (REML) method to account for between-study heterogeneity, which was assessed using the I² statistic, with an I² value greater than 50% indicating substantial heterogeneity.

Sensitivity analyses were performed using the leave-one-out method to assess the robustness of pooled estimates. Publication bias and small-study effects were explored through contour-enhanced funnel plots and Egger’s regression test.

### Quality assessment

The risk of bias was evaluated independently by two reviewers. The Cochrane Risk of Bias tool, ROBINS-I, was used for all included studies. This tool assessed domains including confounding, participant selection, intervention classification, deviations from intended interventions, missing outcome data, outcome measurement, and selection of reported results. Each domain was rated as ‘low’, ‘moderate’, ‘serious’, or ‘critical’ risk, with the overall bias risk for each study determined by the highest risk rating across domains.

## Results

### Search results

**Fig. 1:**
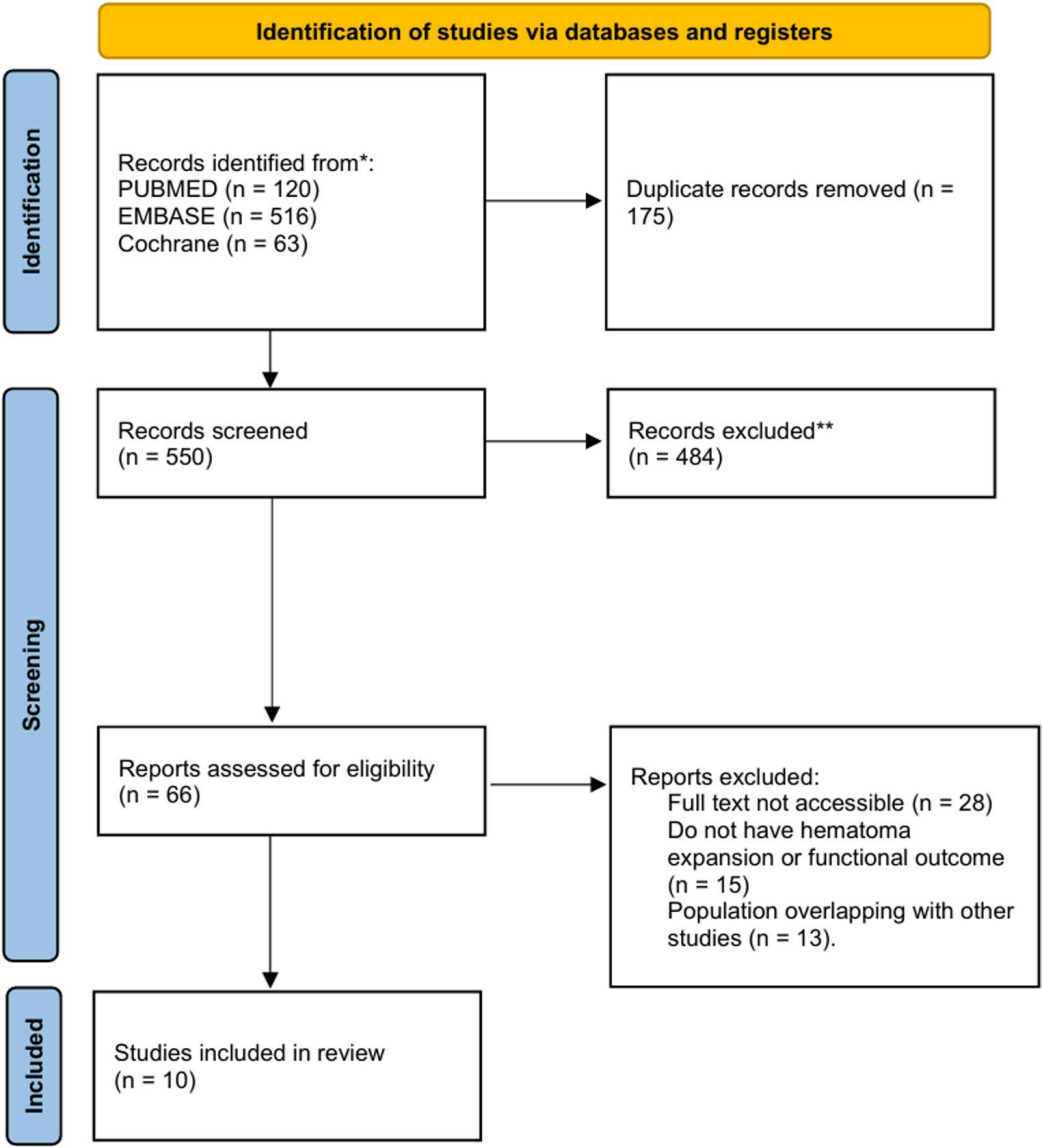
PRISMA Flowchart

The database search, based on predefined inclusion and exclusion criteria, identified 725 articles. A total of 550 studies remained after removing 175 duplicates. Of these, 31 were deemed eligible for full-text assessment, resulting in the inclusion of 10 studies in the review, mostly retrospective cohorts. Figure 1 illustrates the PRISMA flowchart detailing the study selection process and the reasons for exclusion.

### Baseline characteristics

A total of 2,832 patients with spontaneous ICH were included. Of these, 613 patients had SS, while 2219 did not. The mean age was 66.31 ± 13.81. Sex distribution was available for 2,281 patients, of whom 1,367 (58.6%) were male and 914 (41.4%) were female. All characteristics are summarized in Table 1.

**Table 1:** Baseline characteristics of included studies

Study	Design	*N*	M/F	Mean age ± SD	SS/ Non-SS	Onset to CTA time (minutes)
Demchuk, 2012	P	228	130/98	66.12 ± 1.4	61/167	206.3
Falcone, 2022	R	134	94/40	62.3 ± 15.7	18/116	156.1
Han, 2014	R	187	93/94	60.5 ± 14.5	61/126	N/A
Hatim, 2025	R	450	261/189	66.1 ± 12.0	141/309	N/A
Kim, 2025	R	581	347/234	61.6 ± 13.8	70/511	N/A
Moon, 2014	R	287	161/126	61.7 ± 12.7	40/247	N/A
Morotti, 2024	R	551	N/A	72.3 ± 13.4	109/442	N/A
Rosa Junior, 2013	P	65	40/25	58.0 ± 11.9	19/46	650.0
Tseng, 2021	R	66	42/24	55.6 ± 14.9	9/57	N/A
Zhang, 2019	R	283	199/84	60.2 ± 10.7	85/198	N/A

N: number of patients. *M/F* male/female, *SD* standard deviation, *SS/Non-SS* Spot sign patients and non-spot sign patients, *R* retrospective, *P* prospective, *CTA* computed tomography angiography, *N/A* not available

### Functional outcome

**Fig. 2:**
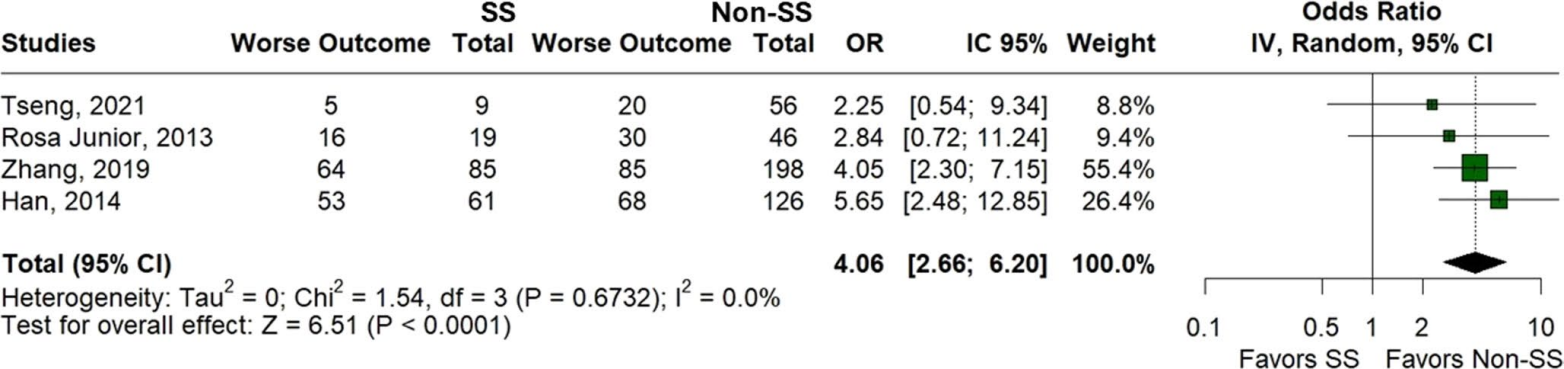
- Functional outcome between Spot sign and non-Spot sign patients

Four studies, including 174 patients with SS and 426 without (*n* = 600), showed a significantly poorer functional outcome in patients with SS compared to those without (OR 4.06, 95% CI 2.66─6.20, *p* < 0.0001) (Fig. 2). There was no substantial heterogeneity among the studies (I² = 0.0%).

The leave-one-out sensitivity analysis (Fig. S1, See Supplementary Data) demonstrated that the pooled effect estimate remained stable after sequential exclusion of each individual study. In all iterations, the association between the presence of Spot Sign and worse outcome remained statistically significant, with pooled odds ratios ranging from 3.61 to 4.30. Heterogeneity remained low across analyses (I² 0.0%), and no single study exerted a disproportionate influence on the overall effect. These findings indicate that the results are robust and not driven by any individual study.

Visual inspection of the funnel plot (Fig. S2, See Supplementary Data) suggested a potentially asymmetric distribution of studies around the pooled effect estimate; however, interpretation is limited by the small number of included studies. Egger’s regression test did not demonstrate statistically significant funnel plot asymmetry (t = − 1.06, *p* = 0.40), indicating no formal evidence of publication bias. However, it is important to note that this test has limited statistical power when only a small number of studies are included. (Supplementary Table 2, See Supplementary Data).

### Hematoma expansion

**Fig. 3:**
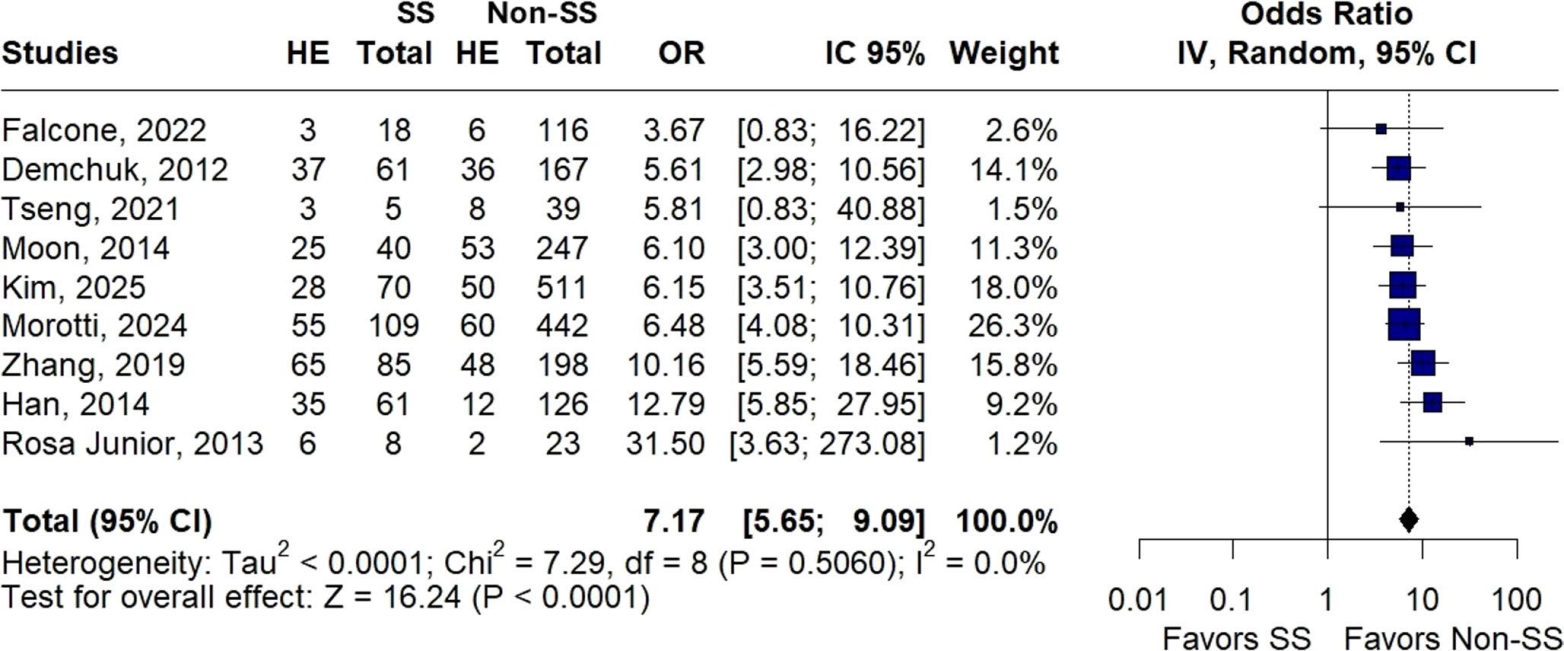
- Hematoma expansion (HE) between Spot sign and non-Spot sign patients

Nine studies, comprising 457 patients with SS and 1869 without (*n* = 2326), indicated a significantly higher incidence of hemorrhage expansion in patients with SS (OR 7.17, 95% CI 5.65─9.09, *p* < 0.0001) (Fig. 3). There was no substantial heterogeneity among the studies (I² = 0.0%). Definitions of hematoma expansion varied across studies, as detailed in Table 2.

**Table 2:** Hematoma expansion definitions across studies

Study	Definition of hematoma expansion
Demchuk, 2012	An absolute growth greater than 6 mL or a relative growth of more than 33% from initial CT to follow-up CT
Falcone, 2022	N/A
Han, 2014	An increase of hematoma volume > 33% or > 12.5 ml
Hatim, 2025	An absolute increase of more than 6 mL or a relative increase of more than 33% in hematoma volume on follow-up CT obtained within 24 ± 12 h
Kim, 2025	An absolute volume increase ≥ 6 cm3 or a relative increase ≥ 33% on follow-up CT
Moon, 2014	An absolute increase > 6 ml or a relative increase > 33% compared with the initial CT image
Morotti, 2024	Defined as volume increase > 66% and/or > 12.5 ml from baseline to follow-up NCCT
Rosa Junior, 2013	An absolute growth greater than 6 ml or a relative growth of more than 33% from the initial NCCT
Tseng, 2021	N/A
Zhang, 2019	Defined as a 33% increase in volume, or an absolute hemorrhage volume greater than 12.5 mL, as determined by follow-up NCCT

*CT* Computed tomography, *NCCT* Non-contrast computed tomography, *N/A* not available

The leave-one-out sensitivity analysis (Fig. S3, See Supplementary Data) showed that removing individual studies did not meaningfully affect the pooled estimate, and the relationship between the Spot Sign and hematoma expansion remained statistically significant throughout (OR range: 6.71–7.46). No single study disproportionately influenced the overall effect. Between-study heterogeneity remained low across all analyses (I² = 0.0%–3.3%), indicating that the observed variability was not driven by any individual study.

Visual inspection of the funnel plot (Fig. S4, See Supplementary Data) revealed an asymmetric distribution of studies around the pooled effect estimate, with a predominance of small studies reporting larger effect sizes and an underrepresentation of studies on the opposite side of the funnel. This pattern suggests the presence of potential publication bias or small-study effects. However, Egger’s regression test did not indicate statistically significant funnel plot asymmetry (t = 0.56, *p* = 0.59), providing no formal evidence of publication bias. However, it is important to note that this test has limited statistical power when only a small number of studies are included. (Supplementary Table 3, See Supplementary Data).

### Mortality outcome

**Fig. 4:**
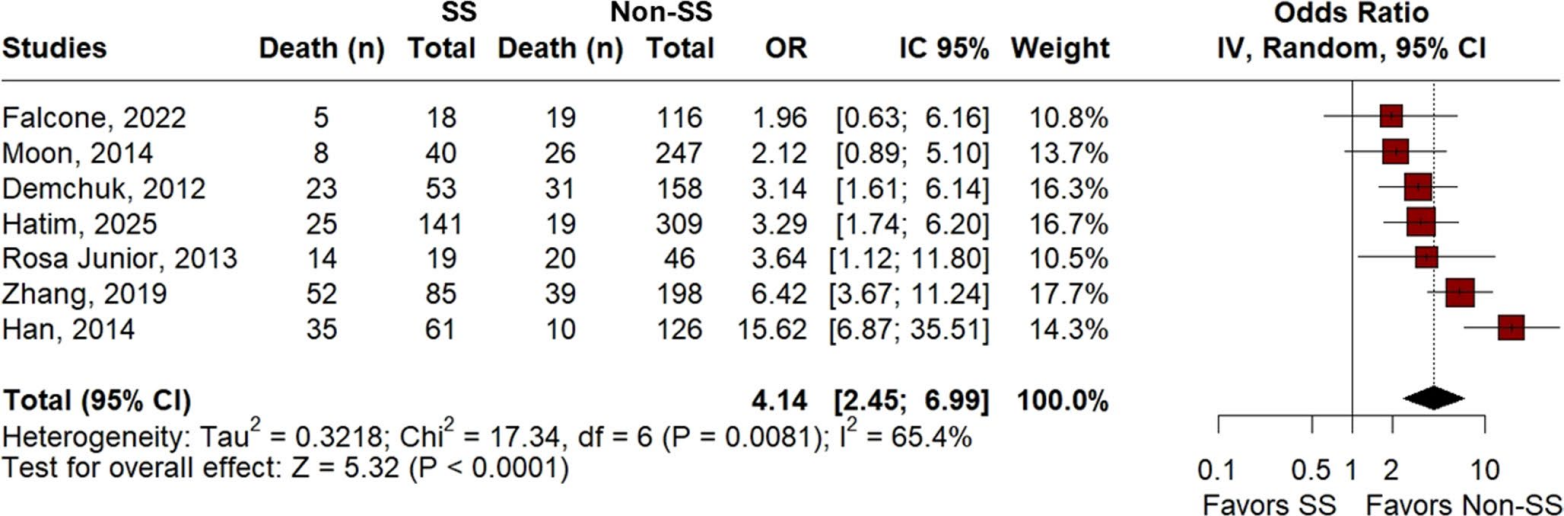
- Mortality between Spot sign and non-Spot sign patients

An analysis of seven studies, involving 417 patients with SS and 1200 without (*n* = 1617), found a significantly higher incidence of mortality in patients with SS (OR 4.14, 95% CI: 2.45–6.99, *p* < 0.0001) (Fig. 4). However, substantial heterogeneity was present among the studies (I² = 65.4%).

**Fig. 5:**
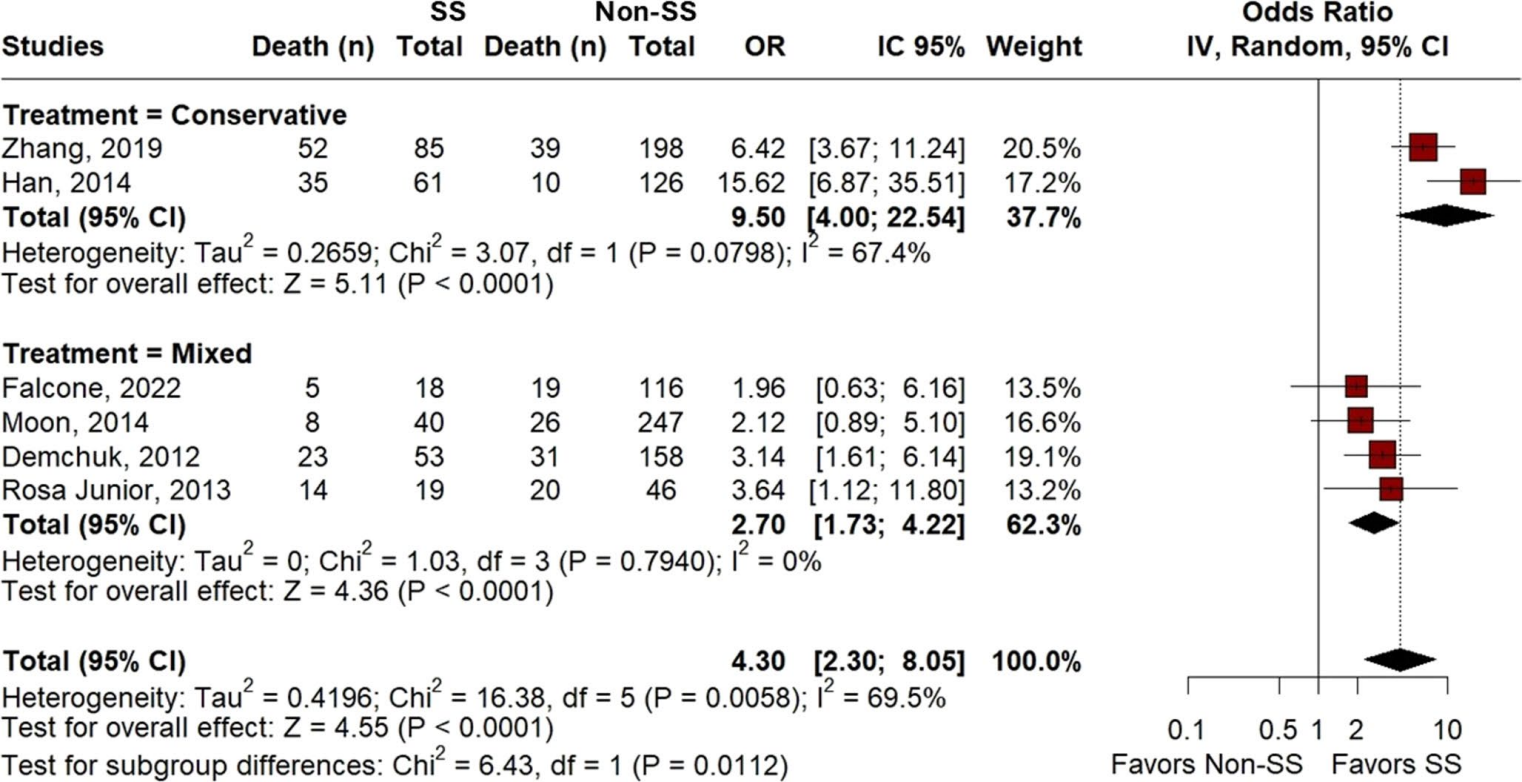
- Subgroup analysis of the mortality outcome by treatment

Subgroup analysis according to treatment modality was performed in studies reporting treatment strategy and demonstrated a statistically significant association between the Spot Sign and mortality in both conservative-only studies (OR 9.50, 95% CI 4.00–22.54, I² = 67.4%) and mixed-treatment studies (OR 2.70, 95% CI 1.73–4.22, I² = 0%) (Fig. 5).

### Initial hematoma volume

**Fig. 6:**
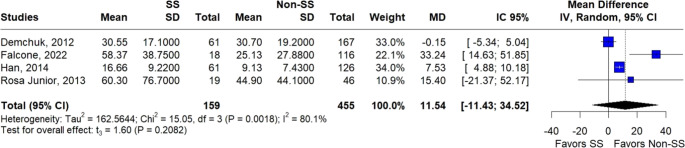
- Initial hematoma volume between Spot sign and non-Spot sign patients

Analysis of four studies, including 159 patients with SS and 455 without (*n* = 614), showed no significant difference in the mean initial hematoma volume between the groups (MD 11.54 mL, 95% CI -11.43–34.52, *p* = 0.2082) (Fig. 6). However, substantial heterogeneity was present among the studies (I² = 80.1%).

### Risk of bias assessment

**Fig. 7:**
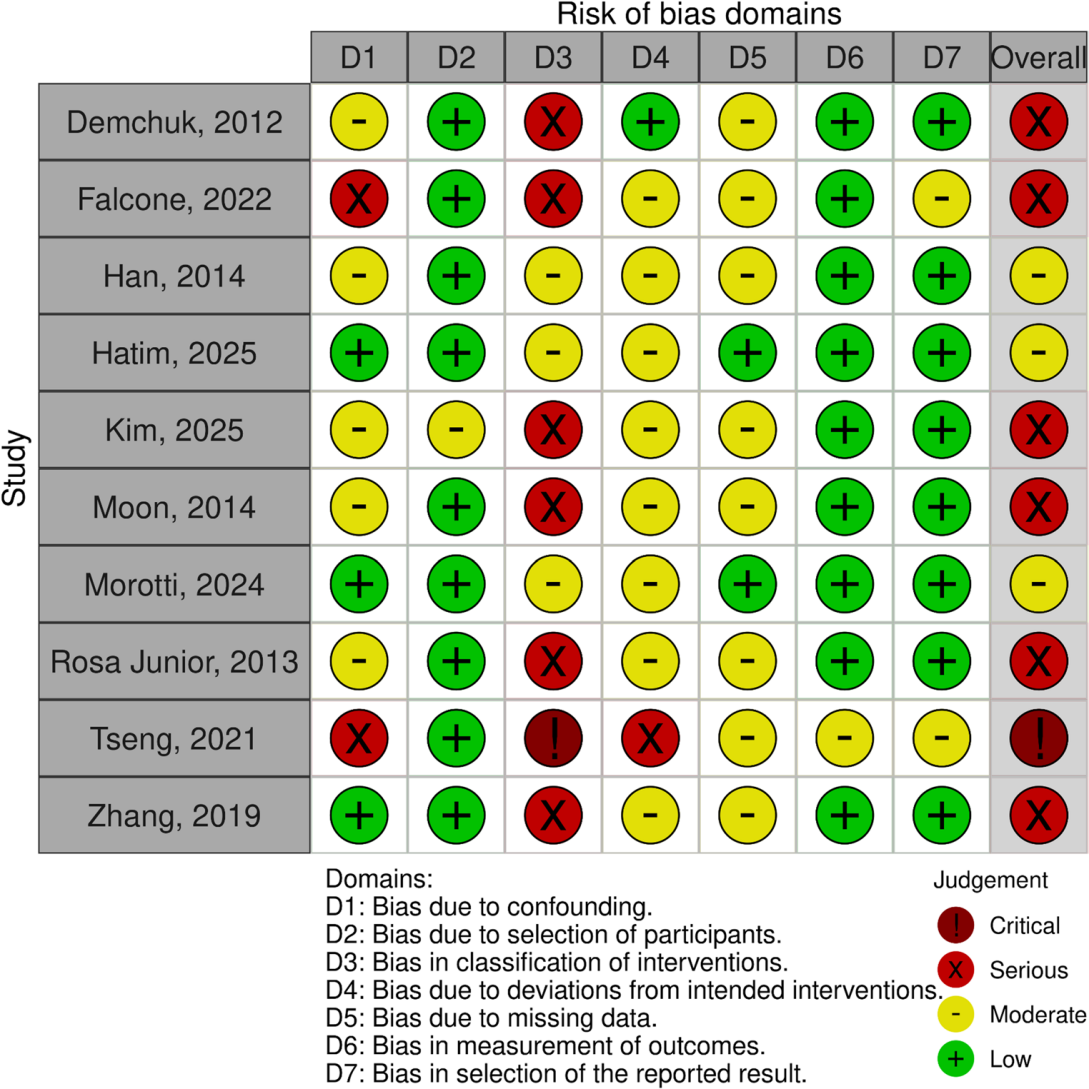
- Risk of Bias Assessment (ROBINS-I)

All included observational studies were assessed using the ROBINS-I tool, demonstrating substantial variability across individual bias domains (Fig. 7). Overall risk of bias ranged from moderate to critical, with the majority of studies judged as having a serious overall risk of bias and no study classified as low risk overall. Bias in the classification of interventions emerged as the most problematic domain, frequently rated as having serious or critical risk of bias. Bias due to confounding was also common, with several studies presenting moderate to serious risk in this domain. In contrast, most studies showed low risk of bias regarding the selection of participants and measurement of outcomes. Deviations from intended interventions and missing data were generally judged as having moderate risk of bias, while the risk of bias related to selection of the reported result was predominantly low. The risk of bias assessment was conducted by two reviewers trained in the application of the ROBINS-I methodology and in accordance with the instrument’s methodological guidance.

## Discussion

In this updated systematic review and meta-analysis including 2,832 patients with spontaneous intracerebral hemorrhage (ICH), we found that the presence of the CTA Spot Sign is strongly associated with adverse clinical and radiological outcomes. Specifically, SS was associated with significantly higher odds of hematoma expansion (OR 7.17), poor functional outcome (OR 4.06), and mortality (OR 4.14). While no significant difference was observed in baseline hematoma volume, the overall findings reinforce the role of the SS as a marker of hemorrhage instability and poor prognosis.

It is important to distinguish primary spontaneous intracerebral hemorrhage from secondary causes, as they differ in pathophysiology, imaging features, and clinical implications. Primary intracerebral hemorrhage is most commonly related to small vessel disease, such as hypertensive arteriopathy or cerebral amyloid angiopathy, whereas secondary hemorrhage may result from vascular malformations, tumors, anticoagulation, or other structural lesions [12].

The SS has been predominantly studied in the context of primary spontaneous intracerebral hemorrhage, where it reflects active contrast extravasation from ruptured small vessels. In secondary hemorrhages, however, the interpretation of this sign is often confounded by underlying structural abnormalities or mimics [13]. Therefore, the interpretation of this radiological sign should take into account the underlying etiology of intracerebral hemorrhage, as its diagnostic and prognostic implications may differ between primary and secondary causes.

The association with hematoma expansion was the most pronounced and consistent finding. The sevenfold increase in expansion risk is biologically plausible, aligning with the understanding of hematoma growth as a dynamic hyperacute process [14]. Notably, statistical heterogeneity was negligible (I² = 0.0%), indicating remarkable consistency across populations and study designs. This reinforces the role of the Spot Sign as a predictive biomarker.

Although the SS is among the most robust predictors of hematoma expansion, variability in its diagnostic performance has been reported. Differences in CTA acquisition protocols, timing of imaging relative to symptom onset, and variation in morphological characterization may contribute to this variability [15–17]. Additionally, quantitative features of the Spot Sign, including number, size, and attenuation (Spot Sign Score), may further refine risk stratification, although these approaches require validation before routine clinical use [18]. These factors highlight the need for standardized imaging approaches to optimize its clinical utility.

Clinically, hematoma expansion is a primary driver of neurological deterioration and long-term disability in ICH [19, 20]. Early identification of high-risk patients may guide closer monitoring, strict blood pressure control, timely reversal of anticoagulation, and selection for hemostatic trials [21]. Although interventional strategies targeting expansion have shown mixed results, accurate risk stratification remains central to individualized management and trial design.

Beyond radiological progression, Spot Sign was associated with a fourfold increase in poor functional outcome, again with negligible heterogeneity (I² = 0.0%). This consistency indicates that the prognostic value of the SS extends beyond imaging-defined progression and into clinically meaningful impairment.

While mortality was also significantly higher in patients with the SS (OR 4.14), the moderate between-study variability observed (I² = 65.4%) likely reflects variations in follow-up duration, regional treatment paradigms, and baseline patient characteristics. Subgroup analysis according to treatment modality demonstrated low statistical heterogeneity among studies including mixed therapeutic approaches (I² = 0.0%), whereas variability remained substantial in cohorts managed exclusively with conservative treatment. Differences in access to neurosurgical care, intensity of neurocritical management, and eligibility for invasive interventions may partially explain these findings. Importantly, the direction of effect was uniform across studies, supporting the identification of SS as a high-risk feature.

Interestingly, the absence of significant differences in initial hematoma volume suggests that the prognostic relevance of Spot Sign is not simply a surrogate for larger baseline hemorrhages. Instead, it appears to reflect hemorrhage dynamics rather than static hematoma size, reinforcing its role as a dynamic biomarker of instability.

The substantial heterogeneity observed in initial hematoma volume (I² = 80.1%) likely reflects both methodological and clinical variability across the included studies. Differences in CTA acquisition protocols, contrast timing, and onset-to-imaging intervals may influence both SS detection and baseline hematoma assessment, particularly since delayed imaging increases the likelihood of capturing contrast extravasation [14]. However, the impact of imaging timing remains difficult to determine, as these data were inconsistently reported. Differences in hematoma measurement approaches, anticoagulant use, and baseline hemorrhage characteristics may have also contributed to the variability observed across studies [19–21].

Compared with prior meta-analyses conducted before 2020 [6–8], the present study updates the available evidence by incorporating larger contemporary cohorts, recent imaging protocols, and additional clinically relevant outcomes. Although our findings are broadly consistent with previous reports, this study provides an updated synthesis of the available evidence while offering additional exploration of potential sources of heterogeneity and clinical applicability. In addition, the present analysis evaluated outcomes that have been less explored in prior pooled analyses, including initial hematoma volume.

From a practical perspective, the Spot Sign remains one of the most extensively studied imaging biomarkers for predicting hematoma expansion and adverse outcomes in acute ICH. Its identification on initial CTA may aid early risk stratification, closer neurocritical care monitoring, and selection of high-risk patients for clinical trials. Nevertheless, despite its strong prognostic value, evidence supporting the role of the SS in therapeutic decision-making remains limited. Clinical trials incorporating the Spot Sign for treatment selection have not reliably demonstrated improved outcomes, suggesting that prognostic relevance does not necessarily translate into therapeutic benefit. Therefore, the SS should be interpreted within a broader clinical and radiological context rather than used in isolation to guide management strategies [22].

## Limitations

Several limitations merit consideration. Most included studies were retrospective observational cohorts, with moderate to serious risk of bias, particularly due to confounding. Differences in baseline hematoma volume, anticoagulant use, and blood pressure management may have influenced effect estimates, and residual confounding cannot be fully excluded. Additionally, variability in outcome definitions and imaging protocols may affect generalizability. Because Spot Sign detection is time-dependent, incomplete reporting of onset-to-CTA intervals may have influenced both detection rates and pooled prognostic estimates. Quantitative and morphological characteristics of the SS, such as number, size, or volume, were not evaluated in this analysis, although previous studies suggest they may provide incremental prognostic value. Sensitivity analyses supported the robustness of the findings, but the limited number of studies reduces the ability to exclude small-study effects. However, this variability likely reflects real-world clinical practice and did not substantially alter the direction of pooled associations. Overall, these limitations should be considered when interpreting the magnitude of effect, although the consistency across analyses supports the interpretation that the SS is a clinically relevant marker of hemorrhagic progression and poor prognosis in spontaneous ICH.

## Conclusion

In conclusion, this meta-analysis demonstrates that the CTA Spot Sign is consistently associated with hematoma expansion, poor functional outcome, and mortality in patients with spontaneous intracerebral hemorrhage. Despite limitations inherent to observational data and the moderate-to-serious risk of bias across included studies, the magnitude and consistency of these associations support the SS as a relevant biomarker of hemorrhage instability and adverse prognosis. Integration of the Spot Sign into early prognostic assessment models may support risk stratification and assist patient selection for future clinical trials. Future studies should further evaluate whether quantitative characteristics of the SS, such as number and size, can refine prognostic assessment and clinical applicability.

## Supplementary Material

Below is the link to the electronic supplementary material.

Supplementary Material 1

## Data Availability

All data supporting the findings of this study are available within the paper and its Supplementary Information.
