# Supplementary material for: Spot sign as a predictor of hematoma expansion and poor functional outcomes after intracerebral hemorrhage: a systematic review and meta-analysis
Source: Emerg Radiol. 2026 May 28;33(4):911–21. doi: 10.1007/s10140-026-02493-z (PMC13433554; doi:10.1007/s10140-026-02493-z)
Supplement: Supplementary file 1 [file 10140_2026_2493_MOESM1_ESM.docx]

*Supplementary material*

Fig. S1 - Leave-one-out sensitivity analysis for functional outcome

Fig. S2 - Funnel plot showing influence and heterogeneity for functional outcome

Fig. S3 - Leave-one-out sensitivity analysis for hematoma expansion

Fig. S4 - Funnel plot showing influence and heterogeneity for hematoma expansion

Supplementary Table 1 - Boolean search strategy

| **Database** | **Search Strategy** |
| --- | --- |
| PubMed | ("Intracerebral Hemorrhage" OR ICH OR "Intracranial Hemorrhage" OR "Cerebral Hemorrhage" OR "intracerebral hemorrhage"[MeSH]) AND ("Spot Sign" OR "Spots Sign" OR Spot-Sign OR “CTA Spot Sign” OR “imaging signs” OR “CTA imaging signs” OR “computed tomography angiography marker" OR "CT angiography marker" OR “CTA marker" OR “CTA sign” OR “CTA imaging sign”) |
| EMBASE | ("Intracerebral Hemorrhage" OR ICH OR "Intracranial Hemorrhage" OR "Cerebral Hemorrhage" OR "intracerebral hemorrhage") AND ("Spot Sign" OR "Spots Sign" OR Spot-Sign OR “CTA Spot Sign” OR “imaging signs” OR “CTA imaging signs” OR “computed tomography angiography marker" OR "CT angiography marker" OR “CTA marker" OR “CTA sign” OR “CTA imaging sign”) |
| Cochrane | ("Intracerebral Hemorrhage" OR ICH OR "Intracranial Hemorrhage" OR "Cerebral Hemorrhage" OR "intracerebral hemorrhage") AND ("Spot Sign" OR "Spots Sign" OR Spot-Sign OR “CTA Spot Sign” OR “imaging signs” OR “CTA imaging signs” OR “computed tomography angiography marker" OR "CT angiography marker" OR “CTA marker" OR “CTA sign” OR “CTA imaging sign”) |

Supplementary Table 2 - Egger test functional outcome

| *Intercept* | *Standard Error* | *t* | *p* |
| --- | --- | --- | --- |
| -1.0190 | 0.9604 | -1.06 | 0.3999 |

Supplementary Table 3 - Egger test for hematoma expansion

| *Intercept* | *Standard Error* | *t* | *p* |
| --- | --- | --- | --- |
| 0.4632 | 0.8200 | 0.56 | 0.5898 |
